# Deep learning classification of early normal-tension glaucoma and glaucoma suspect eyes using Bruch’s membrane opening-based disc photography

**DOI:** 10.3389/fmed.2022.1037647

**Published:** 2022-11-23

**Authors:** Sat Byul Seo, Hyun-kyung Cho

**Affiliations:** ^1^Department of Mathematics Education, School of Education, Kyungnam University, Changwon, South Korea; ^2^Department of Ophthalmology, School of Medicine, Gyeongsang National University Changwon Hospital, Gyeongsang National University, Changwon, South Korea; ^3^School of Medicine, Institute of Health Sciences, Gyeongsang National University, Jinju, South Korea

**Keywords:** Bruch’s membrane opening-minimum rim width, Bruch’s membrane opening-based disc photography, Bruch’s membrane opening overview, deep learning, diagnosis of glaucoma, glaucoma, optical coherence tomography

## Abstract

**Purpose:**

We aimed to investigate the performance of a deep learning model to discriminate early normal-tension glaucoma (NTG) from glaucoma suspect (GS) eyes using Bruch’s membrane opening (BMO)-based optic disc photography.

**Methods:**

501 subjects in total were included in this cross-sectional study, including 255 GS eyes and 246 eyes of early NTG patients. BMO-based optic disc photography (BMO overview) was obtained from spectral-domain optical coherence tomography (OCT). The convolutional neural networks (CNN) model built from scratch was used to classify between early NTG and GS. For diagnostic performances of the model, the accuracy and the area under the curve (AUC) of the receiver operating characteristic curve (ROC) were evaluated in the test set.

**Results:**

The baseline demographics were age, 48.01 ± 13.03 years in GS, 54.48 ± 11.28 years in NTG (*p* = 0.000); mean deviation, −0.73 ± 2.10 dB in GS, −2.80 ± 2.40 dB in NTG (*p* = 0.000); and intraocular pressure, 14.92 ± 2.62 mmHg in GS, 14.79 ± 2.61 mmHg in NTG (*p* = 0.624). Our CNN model showed the mean AUC of 0.94 (0.83–1.00) and the mean accuracy of 0.91 (0.82–0.98) with 10-fold cross validation for discriminating between early NTG and GS.

**Conclusion:**

The performance of the CNN model using BMO-based optic disc photography was considerably good in classifying early NTG from GS. This new disc photography of BMO overview can aid in the diagnosis of early glaucoma.

## Introduction

**FIGURE 1:**
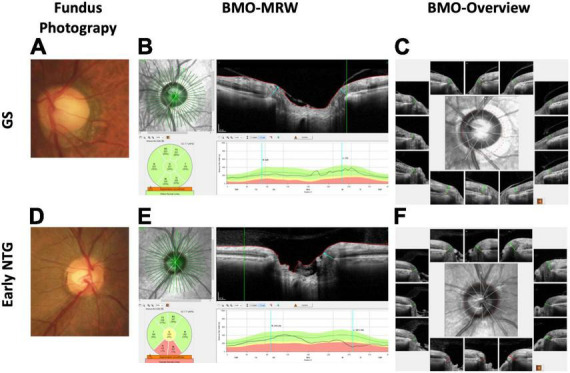
Representative cases of GS and early NTG. **(A)** The optic disc of a 70-year-old male GS patient with a spherical equivalent of −1.0 diopter from fundus photography is shown. Note that the optic disc cup seems big based on the clinical disc margin. **(B)** The BMO-MRW map shows normal color code classification in this GS case. The BMO-MRW is the minimal distance between the inner opening of the BMO and the internal limiting membrane. **(C)** BMO Overview, which is BMO-based disc photography from spectral-domain OCT. It shows the BMO-based disc margin (dotted red line) at the center with 12 cuts around the optic disc, demonstrating each BMO and BMO-MRW at each site. Note that the BMO-based disc margin includes part of the PPA, showing that the optic disc cup does not seem as big based on the BMO margin compared to the clinical disc margin. **(D)** Optic disc of a 56-year-old male NTG patient with a spherical equivalent of +1.0 diopter from fundus photography is shown. Note the thinning of the inferotemporal neuroretinal rim of the optic disc. **(E)**. BMO-MRW map shows abnormal color code classification (red) at the inferotemporal and inferonasal sector in this NTG case. **(F)** BMO Overview shows a shorter BMO-MRW (red lines) at the inferior three cuts compared to the other regions with normal lengths (green lines). Note that the optic disc cup seems markedly enlarged at the inferotemporal region based on the BMO-based disc margin (dotted red line). GS: glaucoma suspect; NTG: normal-tension glaucoma; BMO-MRW: Bruch’s membrane opening-minimum rim width; OCT: optical coherence tomography; PPA: peripapillary atrophy; RNFL: retinal nerve fiber layer.

Glaucoma leads to the damage of retinal ganglion cells (RGC) and their axons, resulting in the deficit of retinal nerve fiber layer (RNFL) and the neuroretinal rim (NRR), which ultimately cause visual field (VF) loss (1). In the diagnosis of early glaucoma, early detection of structural change is more essential than detection of a functional defect (2, 3) since detectable structural change may present in advance of functional VF loss (4–6). As structural damage is minimal in early glaucoma or glaucoma suspect (GS) eyes, differentiate early glaucoma from GS is difficult based on traditional fundus photography alone. As a structural test, optical coherence tomography (OCT) is extensively used in clinical settings and is useful in the diagnosis of glaucoma in early stage. Recently, spectral-domain OCT has been used to provide a new parameter, Bruch’s membrane opening-minimum rim width (BMO-MRW) along with conventional peripapillary RNFL thickness. Moreover, OCT provides BMO-based disc photography, which is called “BMO Overview” by the software. It shows the BMO-based disc margin with 12 cuts around the optic disc demonstrating each BMO and BMO-MRW at each site (Figures 1C,F).

BMO-MRW is the shortest distance between the inner opening of the BMO and the internal limiting membrane (Figures 1B,E). BMO-MRW provides a more precise assessment of the NRR than pre-existing ophthalmic parameters (7–10). Latest studies have shown that BMO-MRW demonstrated better diagnostic performance in glaucoma than preexistent NRR parameters (11–13). In our previous study, we reported that BMO-MRW might reveal normal color code classification, whereas the RNFL showed abnormal color code classification in cases of large discs and myopia (14). This previous study of ours suggested the clinical usefulness of BMO-MRW in early glaucoma or GS, particularly in cases of large disc and myopia when the diagnosis is difficult because conventional color code classification of RNFL may display false-positive results. In another our previous studies using the deep learning method, we reported that our deep learning model using the OCT parameters of BMO-MRW, peripapillary RNFL, and color classification of RNFL provided high diagnostic performance in distinguishing early normal-tension glaucoma (NTG) from GS (AUC, 0.966) (15). Interestingly, as a single parameter, BMO-MRW showed higher diagnostic performance (AUC, 0.959) than RNFL alone (AUC, 0.914) or even RNFL with its color code classification (AUC, 0.934) (15). Moreover, BMO-MRW alone showed diagnostic performance similar to that of all three OCT parameters combined. These findings suggest that the BMO-based optic disc assessment may evaluate different aspects of the optic disc compared to the conventional disc assessments in the diagnosis of glaucoma. To our knowledge, there has been no report of a study using BMO Overview in a deep learning model for the diagnosis of glaucoma.

It is more difficult to differentiate glaucoma of early stage from GS or normal subjects than the glaucoma of advanced stage (16–18). As the field of artificial intelligence (AI) is progressing rapidly these days, the deep learning model may be useful to aid clinicians in this circumstances. Many previous studies have used fundus photography in a deep learning model for the diagnosis of glaucoma (19–25). The diagnostic performance in these previous studies using fundus photography varied, with area under the receiver operating characteristic curves (AUC) of 0.82–0.986 (19–25). Nevertheless, discriminating early stage of glaucoma from GS or healthy is challenging, even using deep learning method, and there are very few studies on early glaucoma. These studies did not include only early-stage glaucoma, and thus, the AUC could vary according to the characteristics of the included subjects. Furthermore, it may be more difficult to discriminate glaucoma of early stage from GS than from a normal healthy subjects.

The prevalence of NTG is higher in Asians than in other ethnicities and NTG is the major type of primary open-angle glaucoma (mean of 76.3%) in Asians (26). Nevertheless, previous studies using deep learning methods for distinguishing glaucoma and normal control rarely included NTG, and studies investigating entirely NTG are hardly found except for our previous study (15).

In this retrospective cross-sectional study, we intended to discriminate early NTG from GS using BMO Overview with a CNN model built from scratch. We evaluated the diagnostic performance and the accuracy of our deep learning model based on convolutional neural networks (CNN or ConvNet). We aimed to investigate whether the new BMO-based disc photography could be useful in the diagnosis of early glaucoma using a deep learning model, which has not been evaluated before. Moreover, there are many previous studies using CNN model with conventional optic disc photography or fundus photography, but none using this new BMO-based optic disc photography. Furthermore, there is no consensus or diagnostic standard for interpreting this new BMO-based imaging, and thus, clinicians cannot examine its diagnostic value, but the deep learning model may aid in this task.

## Materials and methods

### Ethics statement

This retrospective cross-sectional, and observational study was conducted in accordance with the tenets of the Declaration of Helsinki. The present study was approved by the Institutional Review Board (IRB) of Gyeongsang National University Changwon Hospital, Gyeongsang National University School of Medicine. The acquisition of informed consent was exempted from the IRB of Gyeongsang National University Changwon Hospital due to the retrospective nature of this study.

### Subjects

Among a total of 726 patients, 383 patients with normal-tension glaucoma (NTG) and 343 subjects with GS were evaluated between the period of February 2016 and March 2021 in a glaucoma clinic at Gyeongsang National University Changwon Hospital, for a total of 501 eyes (501 subjects) with either early NTG (246 subjects) or GS (255 subjects) were included in the study. All subjects underwent standard ophthalmic examinations, including Spectralis spectral-domain OCT (Glaucoma Module Premium Edition, Heidelberg Engineering, Germany) and standard automated perimetry (HFA model 840; Humphrey Instruments, Inc, San Leandro, CA, USA). Only those subjects who had reliable BMO-MRW and BMO Overview test images and those who met the diagnostic criteria were included. The assessment of early NTG or GS was made by a single glaucoma specialist (H-k Cho) with consistent criteria of diagnosis.

NTG was defined when a patient had an IOP of ≤21 mmHg without treatment presenting findings of glaucomatous damage in the optic disc and corresponding defect in VF, an open angle examined by gonioscopy, and no other underlying cause for optic neuropathy other than glaucoma (27). Early NTG was defined by a mean deviation (MD) of >−6.0 dB on reliable VF tests. Pre-perimetric glaucoma patients were included in the current study to take in the very early stage of glaucoma. Pre-perimetric glaucoma was determined as cases presenting apparent localized RNFL defects on red-free fundus photography with the OCT map of the RNFL confirming the corresponding RNFL defect, but showing within normal limits on Humphrey standard automated perimetry.

GS was determined as those being followed for suspicious clinical characteristics but not definite for glaucoma, such as suspicious optic disc or RNFL changes; significant systemic, ocular, or family risk factors for glaucoma; or suspicious visual field results and intraocular pressure within the normal limits (defined as <21 mmHg on applanation tonometry). None of the GS subjects were receiving treatment for glaucoma by definition and ocular hypertensive patients under treatment were excluded from this study (28). Ocular hypertensive patients who were not receiving treatment were also excluded from this study by the definitive criteria. If both eyes met the inclusion criteria, only one eye was randomly selected.

The exclusion criteria are as follows: poor images due to eye blinking or poor fixation, history of any intraocular surgery aside from uneventful phacoemulsification, history of optic neuropathies except for glaucoma or an acute angle-closure crisis that could affect the thickness of the BMO-MRW or RNFL (e.g., optic neuritis and acute ischemic optic neuritis), and retinal disorders accompanying retinal swelling or edema and consequent BMO-MRW or RNFL swelling. The fellow eyes of unilateral glaucoma were also excluded from the GS group because of the possible effect on BMO-MRW or BMO-based optic disc assessment. Subjects were not excluded from this study by refractive error, axial length, or optic disc size.

### Optical coherence tomography

Imaging of spectral-domain OCT was carried out with Spectralis OCT, Glaucoma Module Premium Edition (Heidelberg Engineering, Germany). Radial B-scans of 24 were acquired for BMO-MRW and BMO Overview. BMO overview image automatically provides BMO boundary points (the red colored dotted line around the optic disc) by the software. Only those images showing well-centered scans and accurate segmentation of the retina and scan quality scores of >20 were taken for the study. Acquirement of data and analysis of OCT scans were conducted employing the individual eye-specific axis (FoBMO axis), which is the axis between the center of BMO area and the fovea. Applying this FoBMO axis could result in more correct analysis of Garway-Heath sectors taking into consideration of the cyclotorsion of individual eyes and thus, lead to more precise comparison to normative database than the traditional means (7). The BMO-fovea angle is the angle between the center of BMO area and the fovea.

### Perimetry

Humphrey Field Analyzer (HFA model 840; Humphrey Instruments Inc, San Leandro, California, CA, United States) were used for perimetry applying a program of Swedish Interactive Threshold Algorithm standard strategy with central 30-2 mode. Reliable VF test were defined with these criteria: a fixation loss of less than 20%; a false-positive rate of <15%; and a false-negative rate of <15%.

### Data preprocessing and dataset

**FIGURE 2:**
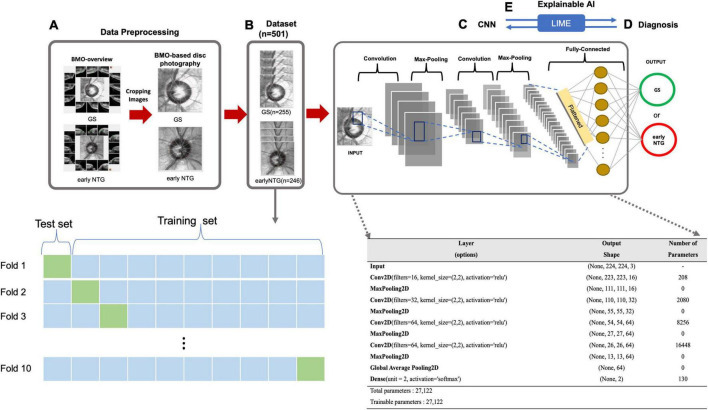
Diagnostic workflow based on the CNN. **(A)** Data preprocessing. BMO-based disc photographs including disc margin (dotted line) were extracted from BMO Overview with a size of 438 × 436 pixels using the Pillow library in Python. **(B)** Dataset. The dataset contains a total of 2 classes with 501 eyes (501 subjects), either early NTG (246 subjects) or GS (255 subjects). 10-fold cross validation was performed to compensate for the relatively small number of dataset. For each iteration, there are 399 images in training set, and 102 BMO-overview images are in test set. **(C)** CNN. A convolutional neural network structure was built from scratch on the Keras Sequential API (https://keras.io/) for the diagnosis of early NTG. The model input was taken as a tensor with dimensions of (244, 244, 3). The first and second hidden layers of the model had 16 and 32 filters, respectively, with a kernel size of (2, 2), and a ReLU was taken as the activation function. The third and fourth hidden layers had 64 filters with a kernel size of (2, 2) and ReLUs. The model contained a fully connected dense layer with 2 units and softmax was taken as its activation function. The batch size was 10, and the number of epochs was 100 in the model. For compiling the model, Nadam (32) and categorical cross-entropy were taken as the optimizer and loss function, respectively. **(D)** Diagnosis. The AI model diagnosed the input images from the test set as either early NTG or GS. **(E)** Explainable AI. Explainable AI (XAI) is artificial intelligence that can be explainable and understandable the predictions or decisions that made by the AI. LIME was applied to understand the decisions of deep learning black-box models. By comparing with the diagnostic criteria of the clinician, the reliability in the diagnosis of the deep learning model can be given.

A total of 501 eyes (501 subjects) with either early NTG (246 subjects) or GS (255 subjects) were acquired from 501 BMO Overview. The dataset consisted of BMO-based disc photographs including the disc margin (dotted line, Figures 1C,F). The BMO-based disc photographs were obtained by cropping the center images from BMO Overview. The cropped center regions were generated with sizes of 438 × 436 pixels using Pillow^1^ in Python 3.7.6, as shown in Figure 2A. Among the datasets, k-fold cross validation (*k* = 10) was performed to compensate for the relatively small number of data set. For each fold iteration, there are 399 images are in training set, and 102 BMO-overview images are in test set. The k-fold cross validation was performed using scikit-learn (sklearn.model_selection.KFold).

### Convolutional neural networks

A deep neural network (DNN) is a well-known supervised classifier containing multiple layers between the input and output layers (29). A convolutional neural network called CNN, which is a type of DNN, is known to have excellent performance in analyzing images (30). A CNN model for classifying GS and early NTG was built on the Keras Sequential API,^2^ written in Python, and running on TensorFlow^3^ (31). In the CNN model for image analysis, tensors of a certain shape were taken as input, and the shape of the tensors was determined by the height of the input images, width, and color channels. Our CNN model used input with dimensions of 244 × 244 × 3 and was composed of 4 convolution blocks. Each convolution block contained a maximum pool layer. The first and second hidden layers of the model had 16 and 32 filters with a kernel size of (2, 2), and a rectified linear unit (ReLU) was applied as an activation function. The third and fourth hidden layers had 64 filters with a kernel size of (2, 2), and applied a rectified linear unit (ReLU) as an activation function. The fully connected dense layer of the model had 2 units with a softmax activation function. The batch size was 10, and 100 was taken as the number of epochs in the model. For compiling the model, Nadam (32) was chosen as the optimizer and categorical cross-entropy was selected for the loss function.

### Explainable artificial intelligence and Local Interpretable Model-agnostic Explanations

AI with Black-box models produce excellent accuracy and diagnostic performances, but it is hard to figure out why they made such a decision (33). Explainable AI (XAI) is artificial intelligence that can be explainable and understandable the predictions or decisions that made by the AI. The XAI algorithm aims for three things: transparency, interpretability, and explanation (34). The Local Interpretable Model-agnostic Explanations (LIME) algorithm is a well-known technique of XAI explaining the predictions of black-box machine-learning models in an interpretable way. It visualizes sections of the image that the CNN model is using to produce its final prediction. The LIME method was originally proposed by Ribeiro et al. (35). The idea of the LIME is that it is easier to interpret for a black-box model to approximate locally by a simpler glass-box. A new dataset containing permuted data and the associated predictions was created, and was used to train the new model, which was weighted by the proximity of the features in the input image to the feature of interest. As the weights were continuously updated, the fully trained new model was used to interpret and predict. Through LIME, the explanations of the predictions of black-box CNN models can be displayed directly on the image samples. The green-colored region indicates that this part of the image increased the probability for the label, and the red color region indicates a decrease in the probability for the label.

### Statistical analysis

Wilcoxon-signed rank test was used to compare the baseline characteristics of the demographic data between the two early NTG and GS and groups for continuous and categorical variables. *p-*values of less than 0.05 were considered to be statistically significant.

## Results

### Baseline characteristics of the datasets

A total of 501 eyes (501 subjects) out of 726 eyes (726 subjects) were included in the final analysis. The GS group included 255 eyes (255 subjects) out of 343 eyes (343 subjects) and the early NTG group included 246 eyes (246 subjects) out of 383 patients (383 subjects). The mean age of the GS subjects was 48.01 ± 13.03 years, which was significantly younger than that of the early NTG subjects at 54.48 ± 11.28 years (*p* < 0.001). The baseline intraocular pressure (IOP) was not significantly different between GS and early NTG, which was 14.92 ± 2.62 mmHg and 14.79 ± 2.61 mmHg, respectively. The mean deviation (MD) of the GS subjects, −0.73 ± 2.10 dB, was significantly higher than that of the early NTG subjects at −2.80 ± 2.40 dB (*p* < 0.001). The pattern standard deviation (PSD) was significantly lower, and the visual field index (VFI) was significantly higher in the GS subjects than in the early NTG subjects (all *p* < 0.001). The central corneal thickness (CCT) was thicker in the GS subjects than in the early NTG subjects (*p* = 0.046). However, the spherical equivalents (SE) were not significantly different between the GS and NTG subjects (*p* = 0.372). The mean SE was −1.93 ± 2.92 D in GS subjects and it was −1.80 ± 2.84 D in early NTG subjects. In GS group, mild myopia (0 to −2.0 D) consisted of 42.7% (109/255), moderate myopia (−2.0 to −6.0 D) comprised 24.3% (62/255), and high myopia (<−6.0 D) comprised 11.0% (28/255). In NTG group, mild myopia (0 to −2.0 D) consisted of 38.6% (95/246), moderate myopia (−2.0 D to −6.0 D) comprised 24.8% (61/246), and high myopia (<−6.0 D) comprised 11.0% (27/246). Approximately 35% of included subjects had more than moderate myopia (<−2.0 D) in both GS and NTG groups. The details of baseline characteristics are demonstrated in Table 1. Forty-three subjects (17.48%) with pre-perimetric glaucoma were included in the early NTG group.

**TABLE 1:** Baseline characteristics of glaucoma suspect and early normal-tension glaucoma subjects.

Characteristics	Values		
Diagnosis	Glaucoma suspect	Early NTG	*P-value*
Number of subjects	255 eyes (255 subjects)	246 eyes (246 subjects)	
Mean Age (year)	48.01 ± 13.03	54.48 ± 11.28	**<0.001**
Female gender (%)	138 (54.11%)	118 (47.96%)	0.147
Family history of glaucoma (%)	16 (6.27%)	27 (10.97%)	0.071
Spherical equivalent (D)	−1.93 ± 2.92	−1.80 ± 2.84	0.372
CCT (um)	546.40 ± 39.18	537.34 ± 60.06	**0.046**
Baseline IOP (mmHg)	14.92 ± 2.62	14.79 ± 2.61	0.624
VFI (%)	98.56 ± 3.94	93.32 ± 6.58	**<0.001**
MD (dB)	−0.73 ± 2.10	−2.80 ± 2.40	**<0.001**
PSD (dB)	2.13 ± 1.33	4.66 ± 2.98	**<0.001**

NTG, normal tension glaucoma; OCT, optical coherence tomography; D, diopters; CCT, central corneal thickness; IOP, intraocular pressure; VFI, visual field index; MD, mean deviation; PSD, pattern standard deviation. Results comparison with GS and early NTG are done with Wilcoxon signed-rank test. Bold font indicates significant *p*-values (*p* < 0.05).

Table 2 demonstrates the BMO-MRW values of the subjects with early NTG and GS. BMO-MRW values of global region were significantly thicker in the GS group than in the early NTG group (262.58 ± 41.32 and 207.42 ± 44.86 um, respectively, *p* = 0.005). The BMO-MRW values from all six Garway-Heath sectors (temporal, superotemporal, inferotemporal, nasal, superonasal, and inferonasal) were also significantly thicker in the GS group than in the early NTG group (all *p* < 0.001). The BMO area in the GS group (2.45 ± 0.52 mm^2^) was significantly larger than that of the early NTG group (*p* = 0.005). Interestingly, the BMO-fovea angle was significantly different between the early NTG and GS groups (*p* = 0.033). The mean BMO-fovea angle was −5.61 ± 3.22° in the GS group and −6.04 ± 3.27° in the early NTG group. This finding indicates that the optic disc was located further away from the fovea in the early NTG group than in the GS group since the BMO-fovea angle is the angle between the BMO center and the fovea. Representative fundus photography of early NTG and GS are demonstrated in Figures 1A,D, respectively.

**TABLE 2:** Brunch membrane opening minimum rim width of glaucoma suspect and early normal-tension glaucoma subjects.

Characteristics	Glaucoma suspect (*n* = 255)	Early NTG (*n* = 246)	*P-value*
BMO-fovea angle°	−5.61 ± 3.22	−6.04 ± 3.27	**0.033**
BMO area (*mm*^2^)	2.45 ± 0.52	2.32 ± 0.59	**0.005**
BMO-MRW G (um)	262.58 ± 41.32	207.42 ± 44.86	**<0.001**
BMO-MRW T	191.42 ± 40.59	162.84 ± 40.18	**<0.001**
BMO-MRW TS	267.62 ± 42.73	207.98 ± 61.80	**<0.001**
BMO-MRW TI	294.68 ± 52.15	192.62 ± 67.87	**<0.001**
BMO-MRW N	275.61 ± 55.89	228.27 ± 58.47	**<0.001**
BMO-MRW NS	291.54 ± 56.98	237.10 ± 63.57	**<0.001**
BMO-MRW NI	320.79 ± 54.85	234.96 ± 64.90	**<0.001**

Values represent mean ± mean deviation. NTG, normal-tension glaucoma. BMO-MRW, bruch’s membrane opening-minimum rim width. G, global. T, temporal. TS, superotemporal. NS, superonasal. N, nasal. NI, inferonasal. TI, inferotemporal. Statistical analysis between glaucoma suspect and early NTG for BMO-MRW was done by Wilcoxon signed-rank test. Bold font indicates significant *p* values (*p* < 0.05).

### Overview of convolutional neural networks model for classifying glaucoma suspect and early normal-tension glaucoma

A CNN model for the diagnosis of early NTG with a convolutional neural network structure on the Keras Sequential API (see text footnote 2) was implemented, as shown in Figure 2.

BMO-based disc photographs including the disc margin (dotted line) from 501 eyes (501 subjects) with either early NTG (246 subjects) or GS (255 subjects) were collected in a glaucoma clinic at Gyeongsang National University Changwon Hospital. The BMO-based disc photographs of 246 early NTG and 255 GS were obtained by cropping the center images from BMO Overview, as shown in Figure 2A. The dataset contained a total of 501 eyes (Figure 2B). 10-fold cross validation was performed to compensate for the relatively small number of data set. For each iteration, there are 399 images are in training set, and 102 BMO-overview images are in test set. The architecture of the CNN built from scratch is demonstrated in Figure 2C. A convolutional neural network structure was built from scratch on the Keras Sequential API (see text footnote 2) for the diagnosis of early NTG. The model input was taken as a tensor with dimensions of (244, 244, 3). The first and second hidden layers of the model had 16 and 32 filters, respectively, with a kernel size of (2, 2), and a ReLU was taken as the activation function. The third and fourth hidden layers had 64 filters with a kernel size of (2, 2) and ReLUs. The model contained a fully connected dense layer with 2 units and softmax was taken as its activation function. The batch size was 10, and the number of epochs was 100 in the model. To compile the model for each CNN, Nadam (32) and categorical cross-entropy were chosen as the respective optimizer and loss function. The AI model diagnosed the images and output as either GS or early NTG, as shown in Figure 2D.

### Diagnostic performances of the artificial intelligence model for discriminating glaucoma suspect and early normal-tension glaucoma

**FIGURE 3:**
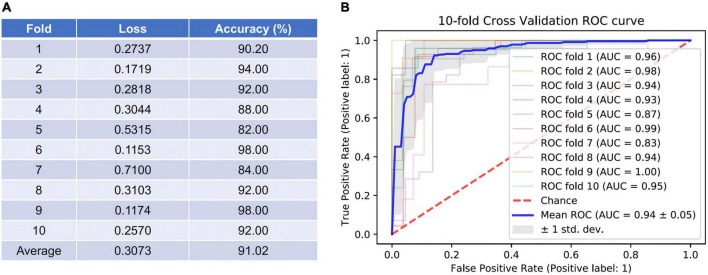
Accuracy and loss per fold and areas under the curve (AUC) for the receiver operating characteristic curves (ROC) achieved by the AI model for classifying GS and early NTG. **(A)** Accuracy and loss per each fold were evaluated. The average loss was 0.3073 (0.1153–0.7100), and average accuracy was 91.02% (82.00–98.00%). **(B)** The area under the curve (AUC) for the receiver operating characteristic curve (ROC) were calculated for the CNN model with 10-fold cross validation. The mean AUC value was 0.94 ± 0.05 (0.83–1.00). GS: glaucoma suspect; NTG: normal-tension glaucoma.

To evaluate the diagnostic performance of the AI model for discriminating early NTG and GS, accuracy, loss, and AUC of the receiver operating characteristic curve over the test set per fold were calculated, as shown in Figure 3. In Figure 3A showed the losses and accuracies for the CNN model with each fold from 1 to 10. In each fold, the number of epochs was 100. The range of loss was from 0.1153 to 0.7100, and the mean loss of the model was 0.3073. The accuracy for the model ranged from 0.82 to 0.98, and the mean average accuracy was 0.9102. The area under the curve (AUC) for the receiver operating characteristic curve (ROC) were calculated for the CNN model with 10-fold cross validation. The CNN model achieved the average AUC of 0.94 ± 0.05 for classifying early NTG and GS in the test set with 10-fold cross validation, as shown in Figure 3B. The highest AUC was 1.00 and the lowest AUC was 0.83.

### Inferotemporal regions were important in classifying glaucoma suspect and early normal-tension glaucoma

**FIGURE 4:**
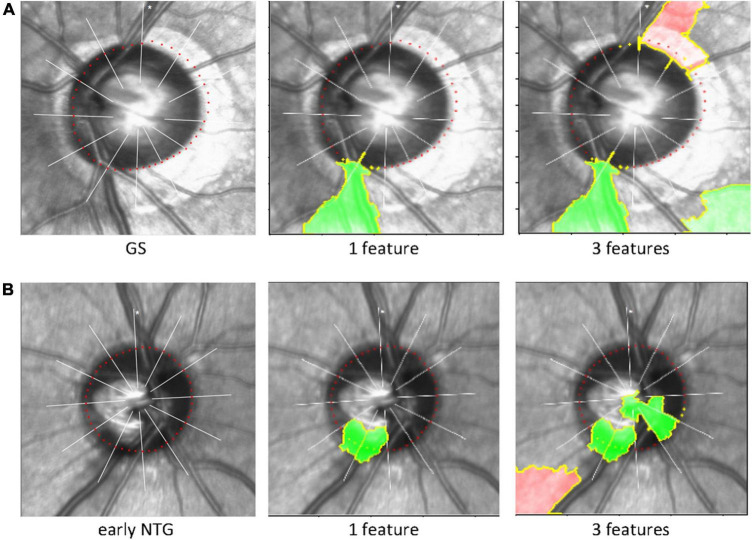
Explainable artificial intelligence and Local Interpretable Model-agnostic Explanations. The LIME algorithm, a well-known technique of explainable AI explaining the predictions of black-box machine-learning models, was used to reveal the area of the image that our CNN model used to extract spatial and temporal features and predict its final classification of the diagnosis (i.e., GS or early NTG). The inferotemporal region of the cupping or neuroretinal rim in the optic disc was considered to be predominantly influential in classifying the final diagnosis. Representative cases are shown for GS and NTG. **(A)** For GS, our CNN model identified the inferotemporal region of the neuroretinal rim (green). **(B)** For early NTG, the inferotemporal region of the cupping and neuroretinal rim were identified for classification (green). Note that the region recognized by LIME (green) includes the BMO points (the red colored dotted line around the disc) in each region for classification of either early NTG or GS. AI: artificial intelligence; LIME: local interpretable model-agnostic explanations; GS: glaucoma suspect; NTG: normal-tension glaucoma.

The black-box deep learning models are generally hard to explain why those made such predictions although they produce great performances and accuracies. The Local Interpretable Model-agnostic Explanations (LIME), a well-known technique of XAI, was used to understand the predicting its final diagnostic classification (i.e., GS or early NTG) of the CNN model in an interpretable way. The LIME algorithm reveals the area of the images that the CNN model used to extract spatial and temporal features. Inferotemporal regions of the cupping or NRR in the optic disc were considered to be predominantly influential in classifying the final diagnosis (Figure 4). Representative cases of GS and early NTG are shown in Figure 4. It shows the extraction of the top 1 and top 3 features, which are the grounds for CNN models to classify GS or early NTG. The green-colored region indicates that this part of the image increased the probability for the label, and the red-colored region indicates a decrease in the probability for the label. In the case of GS, it was mainly determined by the area around the inferotemporal region of the neuroretinal rim (see Figure 4A, green). In the case of early NTG, it was classified as early NTG by the inferotemporal region of the cupping and neuroretinal rim, as shown in Figure 4B (green).

## Discussion

To our knowledge, the current study was the very initial to use BMO-based optic disc photography to discriminate early NTG from GS in a single ethnic group of Asians, where NTG is more prevalent. We found that the diagnostic performance of our CNN model built from scratch was excellent, with the mean AUC of 0.94 (0.83 – 1.00) and the mean accuracy of 0.91 (0.82 – 0.98) in discriminating early NTG from GS. Considering that it is more difficult to classify glaucoma of early stage from GS than glaucoma of advanced stage from normal controls, the results of our study are quite remarkable. Moreover, since there is no consensus or diagnostic standard for interpreting this new BMO-based optic disc photography, clinicians cannot investigate its diagnostic value in the field of glaucoma. Our CNN model has performed this task instead, which will be useful for future research and application in clinical settings.

A previous review article by Sengupta et al. (36) reported glaucoma detection results using deep learning methods with fundus images. The AUC indicating diagnostic performance varied among studies from 0.82 to 0.94, and the highest one was 0.986 (36). Most of these studies used the same CNN model as that in the present study. Some studies showed much lower AUCs than in our study such as 0.82 (24), 0.831 (19), and 0.838 (23). Most of the studies showed AUCs such as 0.923 (25) and 0.926 (37), lower than that in our study, not using CNN, but using Autoencoderut and the feedforward neural network, respectively, and 0.945 (20) using the CNN. Only one study showed a higher AUC than the current study at 0.986 (21) using the CNN. However, these studies did not evaluate only the early stage of glaucoma, which is more difficult to diagnose than advanced stages of glaucoma (16–18). Furthermore, it is more difficult to discriminate glaucoma of early stage from GS than from normal subjects. Considering that our study included only early glaucoma in the discrimination from GS, the AUC results of our CNN model showed fine diagnostic performance. Furthermore, none of these studies included solely NTG for glaucoma nor classified the subtypes of glaucoma as NTG. Thus, the present study has a unique meaning that could add to the existing literature.

Since there is no consensus or diagnostic standard for interpreting this new BMO-based overview imaging yet, clinicians cannot evaluate its diagnostic ability and its value in the field of glaucoma diagnosis. Our newly developed CNN model was able to perform this task and showed that the diagnostic performance of this new BMO-based disc photography was relatively comparable or superior to conventional fundus photography used in most previous studies for the detection of glaucoma. The present study has another significant meaning in this aspect.

BMO-MRW and its BMO overview from spectral-domain OCT have become widely available to clinicians and offer merits rather than conventional optic disc analysis measurements (11–13). BMO-MRW presents a geometrically more precise evaluation of the NRR than preexistent examinations (7–10). BMO-MRW has been reported to be advantageous in correctly reflecting the amount of NRR tissue in the optic disc (38). All of the baseline BMO parameters including the BMO-fovea angle and BMO area showed significant differences between the GS and early NTG groups in our study. The BMO-fovea angle was significantly larger in the early NTG group (−6.04 ± 3.27°) than in the GS group (−5.61 ± 3.22°) (*P* = 0.033). It is a somewhat interesting finding because it means that the center of the optic disc defined as the BMO-based disc margin showed a greater angle from the macula in the early NTG group than in the GS group. Acquirement of data and analysis of OCT were carried out in accordance of the individual eye-specific axis (FoBMO axis), which is the axis between the center of BMO area and the fovea. Using the FoBMO axis could result in a more precise analysis of Garway-Heath sectors regarding the cyclotorsion of individual eyes and more correct comparison with normative dataset than the traditional manner (7). The BMO-fovea angle is the angle between the center of BMO area and the fovea. There has been no previous report regarding the relationship between the BMO-fovea angle and glaucoma, especially in the early stage of glaucoma. The relative location of the optic disc from the fovea is different in each individual and it may possibly affect the development of glaucoma. Retinal nerve fibers or RGC axons could be more stretched and cause more tension in optic discs with a greater angle from the fovea than those with a lesser angle from the fovea. Thus, there could be more conformational change in the optic disc at the lamina cribrosa level in those with a greater BMO-fovea angle than in those with a lesser BMO-fovea angle. However, the spherical equivalents were similar between the GS (−1.93 ± 2.92 D) and the early NTG (−1.80 ± 2.84 D) groups (*P* = 0.372), and patients with relatively mild myopia were included in both groups. Thus, the difference in the BMO-fovea angle between the two groups was not thought to be due to the differences in myopia patients in each group. The association between the BMO-fovea angle and its effect on glaucoma needs to be confirmed in further studies.

The BMO area was significantly larger in the GS group (2.45 ± 0.52*mm*^2^) than in the early NTG group (2.32 ± 0.59 *mm*^2^) (*P* = 0.005). This may be because a large optic disc with a large cup is frequently considered GS (39–42). The BMO-MRW from the global region and all 6 Garway-Heath sectors according to the FoBMO axis were significantly different between the GS and the early NTG group (all *P* < 0.05). The BMO-MRW was significantly thinner in the early NTG group than in the GS group, which indicates glaucomatous changes in the early NTG group and was also reflected in all BMO-MRW regions. The significant difference in all BMO-based parameters including the BMO-fovea angle, BMO area, and BMO-MRWs between the GS and early NTG groups may partly suggest the usefulness of BMO-based assessment in the diagnosis of early glaucoma.

A discrepancy between the clinical disc margin based on fundus photography and the BMO-based disc margin was noted in our study. It has been described in several previous studies, and initially by Chauhan et al. (7, 11, 14). The discrepancy was also noted in our previous study, “Characteristics of Patients Showing Discrepancy Between Bruch’s Membrane Opening-Minimum Rim Width and Peripapillary Retinal Nerve Fiber Layer Thickness” (14). In this previous study, we found that the BMO-MRW may show normal color code classification, while the RNFL is abnormal in GS subjects, especially in patients with large discs and myopia. The discrepancy between the clinical and BMO-based disc margin, in turn, gave rise to discrepancies in the color code classification between the BMO-MRW and the RNFL. BMO-based disc margin takes peripapillary atrophy (PPA) into account. Changes in the optic disc and PPA in myopic eyes were recently described by Sung et al. (43). They found that the morphologic features of the optic nerve head were different based on the β-PPA microstructure in highly myopic eyes (43). Optic nerve head morphology varies among individuals, as does β-PPA. Some patients with β-PPA have basement membrane and some do not. BMO-based disc margin usually includes PPA without BM within the BMO area, which is the BMO-based optic disc area (Figure 1C, GS). This difference in the assessment of the optic disc margin actually affects the assessment of neuroretinal tissue or the neuroretinal rim, which is important in glaucoma diagnosis. The neuroretinal rim seems thinner in fundus photography based on a clinical disc margin without PPA (Figure 1A, GS) than the neuroretinal rim from BMO-based disc photography based on a BMO-based disc margin (Figure 1C, GS), especially in the inferotemporal region with a large PPA in the representative case of GS. Since the variability of optic nerve head morphology and PPA among individuals is partly considered in BMO-based disc photography or BMO-based disc assessment, we assume that the diagnostic performance may be better than conventional assessment in our studies series (14, 15), including this one. Although BMO overview is a black-and white image and does not directly provide values of NRR width (BMO-MRW), it shows BMO-based disc margin considering PPA. Therefore NRR in accordance with BMO-based disc margin can be estimated just like conventional disc photography. Moreover, BMO overview, in fact, provides both clinical and BMO-based disc margin for diagnostic information, which may be more beneficial than conventional disc photography.

Considering the relatively high prevalence of myopia in Asians (26), certain proportion of myopic subjects were included in the present study. Approximately 35% of included subjects had more than moderate myopia (<−2.0D) in both GS and NTG groups in this study. Although we did not exclude any subjects by refractive error or axial length, those high myopic patients whose images were too bad for accurate identification of BMO, and thus, cannot provide accurate BMO-based disc margin were excluded. Several recent studies reported better diagnostic performance of BMO-MRW than conventional peripapillary RNFL thickness in myopic glaucoma patients (44–46). In this regard, our study results suggest that our CNN model using BMO overview may be useful not only in general population, but also in population including considerable proportion of moderate myopia.

We used the LIME algorithm to evaluate the location our CNN model used to classify either GS or early NTG. We confirmed that our CNN model identified the proper region of the optic disc to discriminate between GS or early NTG. Our CNN model identified the inferotemporal region of the optic disc with early NTG with thinning of the inferotemporal neuroretinal rim in the representative case (green area, Figure 4). This may indicate the validity and reliability of the present study since early glaucomatous changes are considered to be initiated in the inferotemporal region of the optic disc (47).

The present study had several limitations. First of all, the retrospective nature of the present study has its potential limitation. Only those who underwent BMO-MRW and BMO overview imaging and had reliable quality in both test images were included in the current study. The influence of such selection of subjects on our results is unknown. Another one is that it was a hospital-based study conducted at a referral national university hospital of the province, and thus, not a population-based design study. Those subjects included in the present study might not represent the whole population. In addition, the current study included only Korean subjects. Our study results regarding NTG, may not apply to other ethnic populations or other glaucoma types. One of the limitations is the relatively small size of sample in the current study that should be considered. Nevertheless, more than 500 subjects with early NTG and GS out of more than 720 subjects were included in the present study and this number was considered to be sufficient to train and test diagnostic performance to distinguish a single disease from single-device data. In order to compensate for the relatively small number of data set, we performed k-fold cross validation (*k* = 10). Through this cross-validation process, all observations (*n* = 501) were used for both training and test, and each observation was used for test exactly once. Therefore, this may be enough to compensate for the limitation of the small dataset, and the results were also considered to be quite good (the mean AUC = 0.94 ± 0.05).

Moreover, the diagnosis discrimination between early NTG and GS included in the dataset was made by one glaucoma specialist (H-k Cho) for more solid and consistent diagnostic standards. Different ophthalmologists may not always draw the same glaucoma diagnosis decision and not all studies were evaluated solely by glaucoma specialists. Baseline characteristics including the VF global indices and all BMO parameters, which are not available in very large datasets of more than thousands of subjects, were also inspected in the present study. Therefore, our data may provide more reliable and consistent results than other deep learning studies with larger numbers of subjects.

In conclusion, the performance of our CNN model using BMO-based optic disc photography from OCT was considerably great in classifying early NTG from GS. This new disc photography of BMO overview can aid in the diagnosis of glaucoma other than conventional disc photography. Our CNN model may be useful in clinical setting for the diagnosis of early glaucoma, which is more difficult than that of advanced glaucoma. A further multi-center study with larger patient numbers is needed to reach ultimate conclusions.

## Data availability statement

The raw data supporting the conclusions of this article will be made available by the authors, without undue reservation.

## Ethics statement

The studies involving human participants were reviewed and approved by Institutional Review Board (IRB) of Gyeongsang National University Changwon Hospital, Gyeongsang National University School of Medicine. Written informed consent for participation was not required for this study in accordance with the national legislation and the institutional requirements.

## Author contributions

Both authors contributed to the design of the study conducted the study, data collection, analysis, management, interpretation, and prepared the manuscript.

## Footnotes

1
https://pillow.readthedocs.io/en/stable/

2
https://keras.io/

3
https://www.tensorflow.org/
